# Supplementary material for: Vital D: A modifiable occupational risk factor of UK healthcare workers
Source: PLoS One. 2024 Apr 16;19(4):e0296247. doi: 10.1371/journal.pone.0296247 (PMC11020869; doi:10.1371/journal.pone.0296247)
Supplement: Attachment — Submitted filename: Response_to_Reviewers.docx [file pone.0296247.s002.docx]

**Response to reviewers**

Dear,

Please find enclosed specific responses that respond to each point raised by the academic editor and reviewer(s).

1. The manuscript has been confirmed to meet PLOS ONE's style requirements to the best of our knowledge.
2. Funding-related text has been removed from the manuscript. The funding statement does not need to be changed.
3. The grant information has been inserted within the funding information section to offer further clarification. No specific grant number exists and managed internally within the hospital trust.
4. The data remains available upon request due to potentially sensitive personal information.
   a. Dr Rengarajan Subramanian remains the data custodian for this work and contactable via email (rengarajan.subramanian@nhs.net).
   b. Data not uploaded for the above reasons.
5. Authorship list in manuscript in-keeping with request.
6. References: reference 23 has been included after review due to added content.

**Reviewer #1 comments:**

- Title: add UK Healthcare workers
  - Change made
- Introduction, paragraph 1: Clarify winter months in the UK (i.e. Canada's winters last at least oct-march/april); expand on signs and symptoms of vit D def
  - Clarified winter months and expanded on signs & symptoms
- Line 35 has an upside down exclamation mark.
  - Removed
- Study population: Clarify the healthcare workers' positions i.e. nurses, docs, porters, respiratory therapists, etc
  - We have expanded upon this in the manuscript
- Line 108: missing period
  - Added
- All tables: I would place percentages behind the value i.e. 42 (6.6%)
  - We have added these to tables were relevant
- Future considerations: Sample same group in winter months and then again at peak summer months
  - No change made to manuscript
- The article would be stronger if the authors expanded on the current literature, such as Sowah et al's 2017 article that was only briefly mentioned. Another suggstion: "Prevention of COVID-19 with oral vitamin D supplemental therapy in essential healthcare teams (PROTECT): protocol for a multicentre, triple-blind, randomised, placebo-controlled trial" by Ducharme et al (2023).
  - We have added to the manuscript based upon this and referenced under [23].

**Reviewer #2 comments:**

- Introduction: Other factors, such as hand hygiene, personal protective equipment (PPE) use, and vaccination status, also play crucial roles in infection prevention for front-line healthcare workers, which must be mentioned in the introduction or discussion section.
  - Content added to manuscript
- Materials and methods: This study was carried out at Mid and South Essex NHS Foundation Trust, and the population does not represent UK front-line healthcare workers. Please modify it appropriately in different parts of the manuscript.
  - Manuscript amended to account for this by referencing a sample of UK front-like healthcare workers.
- The first subsection of the Materials and methods is called study design. However, it is more about sample size calculation and justification.
  - The materials and methods have been restructured based upon this feedback.
- Relevant dates, including periods of recruitment, exposure, and data collection, should be mentioned.
  - Within manuscript
- It is not well clear that the inclusion criteria were for workers at the “Mid and South Essex NHS Foundation Trust” or the National Health Service UK.
  - This has been clarified within manuscript.
- The size of participants from the sampling frame and the proportion who agreed to take part in this study are not mentioned.
  - Participants section amended to clarify.
- Different subsections of materials and methods are mixed up together. Based on the STROBE statement, it could be good to have the following subsections: Study design, Setting, Participants, Variables, Measurements, Data sources, Bias, Study design, Quantitative variables, and Statistical methods.
  - The materials and methods have been restructured based upon this feedback
- Results: Following the STROBE statement for writing and presenting the results could be more appropriate.
  - Results section has been reorganised and amended to align with STROBE statement
- Adding the percentages to the counts in Table 1 makes it more informative, and it is possible to delete the two sentences in Lines 119-122.
  - We have added these to tables were relevant
- It is similar to other tables, and combining Tables 1, 2, 3, 4 and 5 could be reasonable.
  - This has not been changed as it was felt the combined tables was overwhelming when trialled.
- Marking the mean and median in Figure 1 could be informative.
  - This has been added
- Discussion: The authors concluded that vitamin D deficiency/insufficiency puts the front-line UK NHS healthcare Workers at risk of infection with an unfavourable outcome beyond this study's design, measurements and analysis.
  - Corrected within manuscript

**Reviewer #3 comments:**

- Can you specify the reason for Vitamin D deficiency particularly among the front line healthcare workers?
  - This would be beyond the remit of this study to conclude this.
- Does any of the study population had COVID infection during the study period?
  - Active COVID-19 was not tested for during the study. However, it can be assumed that anybody with active COVID-19 at the time of the study would have been self-isolating and therefore not in attendance or included upon study days.
- Does any of the study population was already on calcium supplementation?
  - We established vitamin D supplementation during this study but did not collect data upon calcium supplementation.
- Does any of the study population had CKD or other chronic illness?
  - A range of chronic illnesses were questioned upon within the study questionnaire including CKD, hypertension, diabetes, heart disease, chronic lung disease. A small number (n=3) reported a background of CKD.

All figures have been uploaded and checked through the Preflight Analysis and Conversion Engine (PACE) digital diagnostic tool and uploaded accordingly.

We hope these responses are adequate to proceed.

Your sincerely,

Dr James Phelan & Dr Renga Subramanian.
